# Correction to “Identification of kinesin family member 3B (KIF3B) as a molecular target for gastric cancer”

**DOI:** 10.1002/kjm2.12739

**Published:** 2023-08-11

Yao F‐Z, Kong D‐G. Identification of kinesin family member 3B (KIF3B) as a molecular target for gastric cancer. Kaohsiung J Med Sci. 2020;36:515–522. https://doi.org/10.1002/kjm2.12206

There is an error in Figure 4A. The correct picture that the representive image of tumors isolated from mice is shown below:

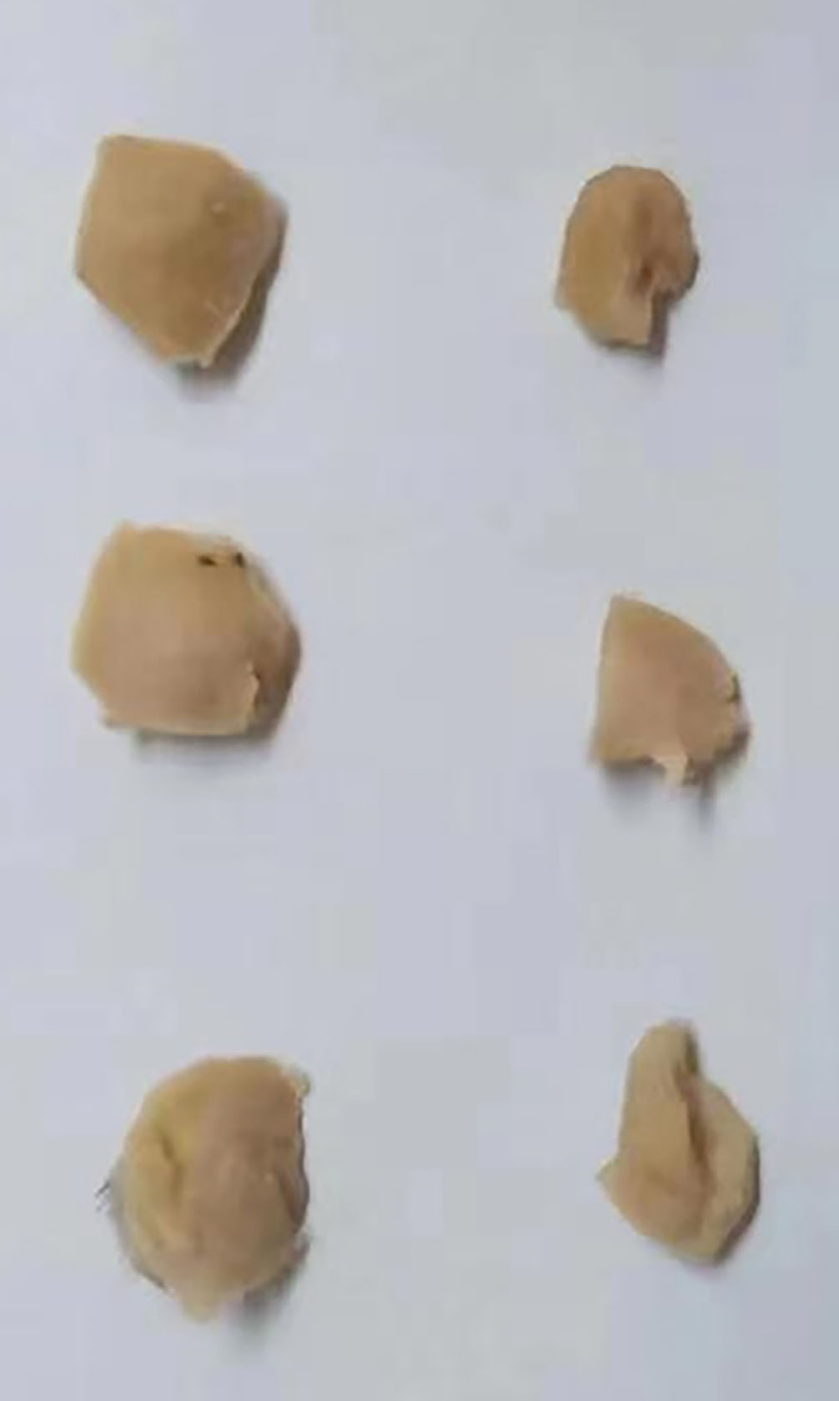

There is a mistake in Figure 4 Part A caption:

Error description: A, MGC‐803 cells infected with control or KIF3B shRNA lentivirus were subcutaneously implanted into nude mice. After 14 days, tumors were isolated, and tumor volume was assessed every 7 days (n = 6 in each group). Left, the representive images of tumors isolated from mice. Right, tumor growth curve was calculated according to the average volume of six tumors in each group.

Correction: A, MGC‐803 cells infected with control or KIF3B shRNA lentivirus were subcutaneously implanted into nude mice. After 14 days, tumors were isolated, and tumor volume was assessed every 7 days (n = 3 in each group). Left, the representive images of tumors isolated from mice. Right, tumor growth curve was calculated according to the average volume of three tumors in each group.

We sincerely apologize for any confusion these errors may have caused and appreciate the opportunity to rectify them.

This correction does not affect the results or conclusions drawn from the data in any way.

